# ‘She come like a sister to me’: a qualitative study of volunteer social support for disadvantaged women in the transition to motherhood in England

**DOI:** 10.1098/rstb.2020.0023

**Published:** 2021-06-21

**Authors:** Jenny McLeish, Maggie Redshaw

**Affiliations:** NIHR Policy Research Unit in Maternal Health and Care, National Perinatal Epidemiology Unit, University of Oxford, Old Road Campus, Old Road, Oxford OX3 7LF, UK

**Keywords:** volunteer, social support, stress, coping, pregnancy, postnatal

## Abstract

This qualitative study explores the ways in which disadvantaged women benefit from social support from a trained volunteer during pregnancy and the postnatal period, using the theoretical frameworks of stress and coping and a multi-dimensional model of social support. Forty-seven mothers took part in semi-structured interviews. The mothers, who had received social support through nine volunteer projects in England, faced many potentially stressful challenges besides having a baby (such as poverty, poor housing, histories of abuse, motherhood at a young age, living with physical or mental health difficulties, migration and insecure immigration status). Analysis was in two distinct stages: first, an inductive thematic analysis of mothers' experiences, and second, mapping of the results onto the theoretical frameworks chosen. Volunteers built relationships of trust with mothers and gave skilled emotional support, positive appraisal support, informational support and practical support according to mothers’ individual needs, thereby assisting mothers exposed to multiple stressors with problem-focused, emotion-focused and perception-focused coping. This helped to reduce social isolation, increase effective access to services and community resources, and build mothers' confidence, self-esteem and self-efficacy. Volunteer social support may have particular salience for mothers who lack structural support and need skilled functional support.

This article is part of the theme issue ‘Multidisciplinary perspectives on social support and maternal–child health’.

## 1. Background

There are significant health inequalities for disadvantaged mothers and their babies in the UK. They are both at increased risk of poor physical and mental health outcomes if the mothers are poor, migrants, from Black, Asian and minority ethnic communities, single or young [1–7]. These outcomes may be influenced by poorer access to maternity and child health services [8] and the effects of stressful life events or chronically stressful circumstances [9,10].

Psychosocial stress can be understood as the interaction between the existence of an objective stressor, a person's subjective cognitive appraisal of the stressor and their reaction in the light of this appraisal [11]. In this model, the experience of stress involves a mismatch, which is the perception that the demands imposed by the stressor exceed the available resources for coping. ‘Coping’ describes behaviours that protect the person from psychological harm resulting from the stressor, and may be focused on removing the stressor, on altering perception of the meaning of the stressor, or on managing the emotional consequences effectively [12]. Some coping resources are psychological, such as a sense of self-efficacy or self-esteem [12]. Another important resource that can buffer the impact of stressors through coping assistance is social support, defined as a person's perception of the availability of others to provide emotional, psychological and material resources [13]. Social support may deal directly with the stressor (problem-focused coping assistance), help to cognitively redefine the stressor as less threatening (perception-focused coping assistance) or alter the person's reactive self-perception (emotion-focused coping assistance) [12,13]. In addition, social support may improve emotional wellbeing by offering companionship, a sense of belonging and mattering to other people [14].

Mothers typically receive social support from their partners, family (especially their own mothers), friends and health professionals when they have a baby. Social support has been found to enable mothers to cope more effectively with the stress of having a baby [15,16], and it is an important protective factor against antenatal and postnatal depression and anxiety, and a key ingredient for developing parenting confidence [6,16–22]. Social support is constructed as both *structural*, which refers to the number of relationships in a person's social network, and *functional*, which refers to dimensions of the support itself—emotional, appraisal (esteem), informational and practical [23]. Emotional support, which contributes to emotion-focused coping, consists of words or actions that show love, liking, empathy, respect and trust, leading the recipient to believe that they are cared for, esteemed and valued [23]. Appraisal support, which contributes to perception-focused coping, is the communication of information to enable positive self-evaluation [23]. Informational support, which contributes to problem-focused coping, is information provided to another at a time of stress [23]. Practical support, which also contributes to problem-focused coping, is the provision of tangible goods, services or aid [23]. These different dimensions of social support operate through multiple social psychological mechanisms [14]. Different people in the network may provide different dimensions of functional support and a mother may feel unsupported or even more stressed if the support offered does not match her needs [13,15,21].

People living with various forms of disadvantage are disproportionately likely to be affected by stressful events [24]. They are also less likely to have the psychological and interpersonal resources to cope, because of absent or limited social networks and lower self-esteem and self-efficacy [25,26]. Where a mother does not have sufficient social support from within her own family and social network, she may benefit emotionally from support from a trained community volunteer [27–34]. There is as-yet limited research on the specific dimensions of social support during the transition to motherhood [35]. This paper aims to explore the experiences of disadvantaged mothers who received social support from a volunteer, through the lens of the theoretical framework of stress and coping and a multi-dimensional model of social support.

## 2. Methods

This qualitative study in two stages was informed by the theoretical perspective of phenomenological social psychology [36]. The first stage had an experiential qualitative descriptive design [36,37], which focuses on mothers' accounts and enabled their voices to be heard while acknowledging the role of both participants’ understandings and the researchers' interpretations in the production of knowledge [38]. Results of the empirical stage have already been reported [39–41]. The second stage was theory-based mapping of those qualitative data, reported here.

### (a) Participant recruitment

A researcher (J.M.) contacted the coordinators of nine projects providing volunteer social support to disadvantaged mothers in England. Each project trained unpaid volunteer mothers from the local community in active listening, providing information and signposting to local services. Volunteers supported women during pregnancy and until the baby was between six weeks and two years old, through one-to-one support, group support or both. The projects had a variety of target groups, including young mothers, South Asian mothers, refugee and asylum seeker mothers, mothers living with HIV, mothers with mental health difficulties, mothers with very complex needs or any disadvantaged mother in the local area. Some volunteers were peers with specific life experiences as well as motherhood, such as living with mental health difficulties or HIV. Details of the projects have been reported in an earlier paper describing the different models of volunteer support [40]. The project coordinators described the research to supported mothers using the study information leaflet and either asked permission for the researcher to contact them or arranged with those who wished to participate a time for interview.

### (b) Data collection

Each mother took part in a face-to-face semi-structured interview that explored the mother's experiences of using maternity services, her involvement with the volunteer support project, its impact, and her feelings about the voluntary nature of the peer support and its ending. The duration of interviews varied (range 16–90 min, median 44 min); the shorter length of a few interviews was owing to mothers needing to attend to their young children. Professional interpreting for participants whose first language was not English was offered, but none took up the offer. All the interviews were audio-recorded and fully professionally transcribed, with each participant being given an anonymous identifier, e.g. M001.

### (c) Data analysis

Interviews were initially analysed using inductive thematic analysis [42]. Transcripts were checked against the audio recording, and then reread, and codes were identified inductively and recorded using NVIVO software. Codes were refined, combined and disaggregated as data collection continued, and themes identified; initial codes and themes were reconsidered in the light of subsequent interviews. To increase the validity of the analysis, one researcher (J.M.) undertook thematic analysis of all the transcripts and the other (M.R.) analysed a subset. Codes and themes were discussed and agreed. Both researchers approached the analysis reflexively, acknowledging the potential impact of their own perspectives as White, UK-born women with children. After this inductive analysis, a second stage of analysis was carried out deductively: the themes identified were mapped onto the theoretical framework of stress and coping theory and a multi-dimensional model of social support.

## 3. Results

### (a) Participants

A total of 47 mothers who had received volunteer support during pregnancy and after birth took part in interviews between July 2013 and September 2014. Forty-six interviews were carried out face-to-face and one interview was by telephone at the mother's request. Mothers’ socio-demographic characteristics are shown in table 1. They all faced a range of challenging issues and most faced multiple challenges. These stressors included traumatic experiences before pregnancy, such as forced migration (to seek asylum or through people trafficking), having children taken into care, the death of a child or partner, childhood sexual abuse, detention, rape and torture. Ongoing stressful experiences during pregnancy and afterwards included unemployment, poverty, homelessness, domestic abuse, children with health or behavioural problems, living with stigma (for example because of HIV), unfamiliarity with the UK healthcare and social support systems, language barriers with service providers and insecure immigration status. Other stressors related to the mother directly, such as pregnancy at a young age, poor physical or mental health, and living with HIV. Finally, for some mothers, there were stressors specifically connected to motherhood: a lack of knowledge about pregnancy, birth and parenting, and adapting to the role transition of becoming a mother.

**Table 1.** Characteristics of mothers interviewed.

	number of mothers (*n* = 47)	percentage (%)
first time mother	22	47
aged under 25	10	21
single parent	21	45
long-term health condition or disability	9	19
poor mental health^a^	25	53
ethnicity		
Black	18	38
White	13	28
Asian	3	6
Other	8	17
born overseas	31	66
English not first language	35	74
asylum seeker/refugee	13	28

^a^This refers to mothers' self-described poor mental health during pregnancy or after birth.

### (b) Findings

Table 2 shows the theoretical mapping of the stressors and support activities described by mothers in the empirical stage [39–41], and the social, psychological and practical mechanisms of impact identified by the authors during that stage, with illustrative quotations.

**Table 2.** Stressors and support activities mapped onto the dimensions of social support and coping theory.

stressor	support activity	aspect of social support	coping	mechanism identified	example quotation
social isolation	regular contact	structural/companionship	problem-focused	increase in social network	‘If she wasn't there I would feel like alone, crying every day’ (M010)
		emotional	emotion-focused	feeling valued	*‘*It's something inside yourself as well, you know that that person is there in their own time and there because they want to be, not because they have to be’ (M016)
	signposting or accompanying to local parent groups	informational, practical	problem-focused	increase in social network	‘She helped me get into the baby groups and get me out the house a bit more, which built my confidence up’ (M026)
no-one to confide in	non-judgemental active listening	emotional	emotion-focused	offloading, feeling heard	*‘*When the problem is really, really much I feel depressed, I just smash everything on her and she listens to me’ (M028)
				feeling accepted	‘You can be open and you can be yourself, and if you have something on your mind you know you can say it without being judged’ (M019)
chronic and uncontrollable stressors	compassionate presence, moral support, shared prayer	emotional	emotion-focused	having someone alongside	‘The emotional stress … you might say, “Oh God, I did not bargain for this” … When someone is there to encourage you I think you will feel better’ (M028)
lack of confidence, feeling powerless, feeling like a failure	normalization	appraisal	perception-focused	lateral social comparison	*‘*They took my mind off a lot of what I was going through, the problems and worry, by making feel me like I was normal’ (M007)
	strengths-based approach	appraisal	perception-focused	affirmation	‘She made me feel better because [she was] speaking always good things [about] me’ (M013)
	encouraging small steps towards goals or solutions	appraisal	perception-focused	building experiences of success—increasing self-efficacy	‘She used to ask me, “What do you want to do? You can do it! …” I have always been living toward the things we wrote down to achieve it’ (M006)
	non-directive information	appraisal	perception-focused	empowerment	‘The volunteer provides a package of solutions, choice, and they told you what's pros and cons, and you make decision which is right for you. There is no push, no demand’ (M043)
lack of hope	role modelling recovery or positive outcomes	appraisal	perception-focused	inspiring hope	*‘*I'd be like, “Oh wow, they're normal now, and that's going to be me”’ (M038)
negative interpretations	suggesting alternative explanations	appraisal	perception-focused	reframing	*‘*They show me another way of looking at it, where sometimes I'm very narrow-minded’ (M019)
lack of knowledge about pregnancy, birth, parenting	providing evidence-based information	informational	problem-focused	empowerment, confidence	‘She told me everything … When I went for labour, when I gave birth, when I had a baby, it was like it wasn't new to me’ (M032)
poverty, hunger	signposting to local services, community groups, food banks	informational	problem-focused	meeting basic needs	*‘*When I was pregnant, she make me a lasagne. Oh, I'll never forget this lasagne! … She brings me clothes for the baby before she [was] born. She showed me [the community centre], they give free baby clothes and free baby stuff’ (M010)
	providing baby clothes, household goods, food	practical			
unfamiliar systems	explaining systems, signposting	informational	problem-focused	effective access to services	*‘*You have to try to find things, how they're going to work … You always need someone who can explain for you. And who knows the law’ (M037) ‘I wouldn't want to go on my own … I was like, “Oh God, I can't do it, I can't do it.” And she's like, “Just go. I'll come with you’’’ (M020)
	accompanying, advocacy	practical			
communicating with health professionals	interpreting, advocacy	practical	problem-focused	effective use of services	‘She came to nearly all my appointments … it's much better because sometimes I can't explain myself because my English, I can't remember all the words’ (M011)

#### (i) Structural support and companionship

Most of the mothers were extremely socially isolated. Sometimes, this was as a result of migration to the UK or because of homelessness and temporary accommodation: ‘I had so many difficulties here because I was so alone here’ (M036). Others had structural support in theory from partner, family or friends, but could not make use of it because confiding their difficulties would trigger negative reactions: ‘I can't tell people I can't cope. In Africa they would say, “Then why did you get pregnant?”’ (M006). The volunteers represented an increase in mothers' social networks, and their support included helping mothers to access local parenting groups where they could find ongoing structural social support.

The significance of this companionship was reflected in the dominant metaphors used by mothers to describe their volunteers, which were either social: ‘Friends forever, friends for life!’ (M001) or echoed the family relationships they were missing: ‘I have a home’ (M033); ‘She come like a sister to me’ (M012). Although some mothers moved on naturally from needing this additional structural social support, many preferred that the support relationship ended by evolving into informal social contact. Where a project had a defined endpoint and withdrew the volunteer from contact at the end, this loss of structural support could be distressing for mothers: ‘Hard … she's been like part of the family almost’ (M030). The relationship of trust built by the volunteers over time was the vehicle for effective delivery of all four functional dimensions of social support.

#### (ii) Emotional support and emotion-focused coping assistance

Visits from an unpaid volunteer gave vulnerable mothers the sense that they had an individual social value. All the volunteers were trained in the techniques of active listening, and their non-judgemental demeanour enabled mothers to speak freely, unburden themselves of thoughts they had been keeping to themselves, and experience unconditional acceptance. Having difficult feelings accepted and validated helped mothers to accept themselves. Where the mother was dealing with a chronic stressor that could not be solved, such as insecure immigration status, volunteers gave moral support through compassionate presence, solidarity and prayer.

#### (iii) Appraisal support and perception-focused coping assistance

Volunteers used multiple techniques that enabled mothers to appraise themselves and their situations more positively, including drawing on their own peer experiences of motherhood (or other challenging issues) to normalize the difficulties and emotions mothers were experiencing. In helping mothers to reframe how they saw themselves and their competence, most volunteers used an explicitly strengths-based approach that affirmed mothers' capabilities, and supported them in taking small steps in dealing with stressors. They gave non-directive information rather than advice, empowering mothers to make their own choices by emphasizing the mothers’ agency. They gently challenged mothers' self-blame and negative interpretation of interactions. Volunteers with direct peer experiences in addition to motherhood also provided role models of recovery or living successfully as a mother with the condition or in the situation, inspiring mothers with hope for their own future.

#### (iv) Informational support and problem-focused coping assistance

Volunteers were trained to give evidence-based information about pregnancy, birth and parenting that was not based on their own experiences but learned during training or obtained from reputable sources. They might draw on support from their project or their own knowledge to help mothers understand the UK maternity care and welfare systems, resources available in the local community (such as a parenting group, a free source of baby clothes, a food bank or a place of worship) and life in the UK (for example, bus routes, or how to use a self-service checkout).

#### (v) Practical support and problem-focused coping assistance

Volunteers did not just give mothers information about community resources and UK systems, but in many cases, gave them direct practical help to make use of them. This was typically in the form of accompanying an unconfident mother to the group or service, advocacy on her behalf to service providers, or informal interpreting so she could make effective use of the service. Some volunteers mobilized their own networks to provide mothers with essential items for the baby or household goods that they lacked, or took them home-cooked food.

## 4. Discussion

Studies of social support in the transition to motherhood often focus on support for the specific challenges associated with having a new baby, usually for more advantaged participants [16]. Although the volunteer projects in this study offered support for women during pregnancy and afterwards, having a baby was just one of a range of stressors affecting the disadvantaged mothers from a range of cultural backgrounds. Mothers described the benefits when volunteers gave support in ways that also addressed these wider contextual stressors, flexibly adapted to a mother's individual needs. Successful volunteer support for mothers has been consistently found to be based on a relationship of trust [27,28,40,43–46]. Mapping the types of support given by volunteers onto the four dimensions of social support and the three types of coping assistance indicates the breadth of support that can be given by volunteers within this relationship, using a variety of techniques.

From the perspective of stress and coping theory, this study illustrated how volunteers gave multiple forms of coping assistance. They helped mothers directly with some of the problems causing them stress, for example, by helping them to navigate UK systems, make more effective use of maternity care, find community resources and access essential items for their babies and emergency food. Emotion-focused coping took the form of providing mothers with a compassionate and confidential listener to whom they could offload their worries and stress, and making them feel that they mattered and were not alone as they faced their difficulties. As predicted by Cutrona & Russell [47], this emotional support was central when the stressors themselves were uncontrollable. Some of the most skilful interactions were forms of perception-focused coping in which volunteers reframed mothers' perceptions of situations and also of themselves and their ability to cope. Many mothers described themselves in ways consistent with very low self-esteem and self-efficacy. These self-perceptions were modified over time, with mothers gradually feeling themselves worthy of attention and care. The volunteers’ presence and gentle affirmation, their non-directive information-giving emphasizing the mothers' own decision-making, support for achieving small goals, and opportunities to feel normal and hopeful about the future through social comparison, actively contributed to this process [48].

Mothers normally rely on structural social support from their existing social network, but most mothers in this study had low levels of structural support, or none at all, which is particularly common for migrant women [49]. Volunteers supplied a basic level of structural and companionship support through their visits, and helped to build up mothers’ social networks by providing groups for them to meet each other, or by connecting them to other community sources of structural support such as parent and child groups. The importance, and sometimes challenge, of managing the ending of support sensitively [27] was emphasized by mothers' frequent use of metaphors of family and home when describing their personal experience of receiving volunteer support, particularly when their real family or home had been left behind or lost.

Mothers who have structural support may be dissatisfied with the level or type of functional support they receive from their network, and support may be ineffective (e.g. wrong advice) or may be counterproductive if it does not match need (e.g. perceived as controlling or undermining), which can increase stress and contribute to poorer psychological wellbeing [6,16,50]. Some mothers in this study had some apparent structural support but did not see their partner, family or friends as safe and useful sources of functional support, particularly if they were ashamed of their situation or feelings, or were worried about the consequences of being perceived as not coping [41]. This is in line with the personal and social barriers to mobilizing postnatal social support identified by Negron *et al*. [51] and De Sousa Machado *et al*. [52], who note that there may be cultural differences in these barriers. Although the level of training varied between the projects [40], all the volunteers were trained in non-judgemental active listening, confidentiality, non-directive information-giving and a strengths-based approach to building up mothers’ confidence and self-efficacy. This meant that, unlike partners, family and friends without the benefit of training, volunteers were able to give effective social support across all the functional dimensions of social support: emotional, informational, appraisal and (in many cases) practical support as well. This echoes the findings of a study of social support from paid lay pregnancy outreach workers [53].

A key strength of this research was the inclusion of 47 participants with diverse personal circumstances, including some extremely disadvantaged backgrounds, from nine different volunteer peer support projects around England. Another strength was the separation of the deductive theoretical analysis from the initial inductive stage, so that analysis first stayed close to participants' own perspectives and words before the later application of a theoretical framework [36]. There were also some limitations. First, participants were contacted through the project coordinators—this was essential to gain the trust of vulnerable women, but meant that the researchers were not aware of how many declined to participate at that early stage. Second, one mother's interview was informally interpreted by her volunteer supporter at her request, so her comments about the support she had received had to be considered in this context (she is not quoted in this paper).

## 5. Conclusion

Volunteer social support during pregnancy and after birth is a promising and valued intervention that can benefit disadvantaged mothers through a number of interrelated mechanisms. Trained volunteers can give emotional, appraisal, informational and practical support according to mothers' individual needs, thereby assisting mothers exposed to multiple stressors with problem-focused, emotion-focused and perception-focused coping. Evidence from this study indicates that volunteer social support has particular salience for women who lack structural support and have complex needs that are not easily met within conventional care, for example, mothers who are recent migrants and those experiencing multiple complex disadvantages.

## Data accessibility

Women consented to data collection and for their interview responses to be used in publications with no details or other information being published that could identify them. Following the consent process, the individual qualitative interview transcripts will not be made publicly available.
